# Development of the Sri Lankan Early Teenagers' Violence Inventory: An Instrument to Measure Peer Violence in Schools

**DOI:** 10.1155/2014/563143

**Published:** 2014-06-29

**Authors:** Monika Wijeratne, Rohini Seneviratne, Nalika Gunawardena, Truls Østbye, Catherine Lynch, Ingvild Fossgard Sandøy

**Affiliations:** ^1^Ministry of Health, Colombo 01000, Sri Lanka; ^2^Department of Community Medicine, Faculty of Medicine, P.O. Box 271, 25 Kynsey Road, Colombo 00800, Sri Lanka; ^3^Duke Global Health Institute, Duke University, P.O. Box 90519, Durham, NC 27708, USA; ^4^University of Bergen, Centre for International Health/Department of Global Public Health and Primary Care, P.O. Box 7804, 5020 Bergen, Norway

## Abstract

This study was designed to develop an inventory to measure peer violence among early teens (13–15 years of age) in schools in Sri Lanka. Development of SLETVI was carried out in two phases. In phase I, development of an operational definition for peer violence, identification, and finalizing violent acts for inventory was done by a combination of qualitative methods: a comprehensive literature review, focus group discussions among 13–15-year-old adolescents, their teachers and parents, and consultative meetings with experts in the field. Inventory was then pretested. In phase II, elaboration of SLETVI was carried out by administering it to a sample of 1700 adolescents (13–15 years old). Exploratory factor analysis using principal component analysis was performed separately for experiences of victimization and perpetration. Test-retest reliability of SLETVI was assessed. SLETVI included 37 items in three factors: “less severe violence,” “severe physical,” and “severe relational” violence. Combined use of qualitative and quantitative methods enabled development of a culturally valid and reliable operational inventory to assess early teenagers' peer violence in Sri Lankan and other South Asian schools.

## 1. Introduction

According to the national survey on emerging issues among adolescents (*n* = 29,911) in Sri Lanka, 75% of participants had experienced some form of peer harassment in school [1]. Harassment or violence in schools adversely affects the physical and mental health of adolescents, and thereby it disturbs their education. To assess the magnitude of the violence in schools, scientific measurements and accurate data are essential. Depending on the purpose of the study, various sources of data can be used to measure violence in schools. One approach is to use morbidity and mortality data, usually recorded by the police or hospitals. Only the extreme or severe cases are likely to be captured by this procedure [2]. In some countries, school statistics are available on violence when there is a systematic method of record keeping. Unfortunately, in Sri Lanka and other South Asian countries such surveillance systems are not available. For this reason school based surveys are likely to be the best available option to capture the spectrum of violence among students.

Violence among adolescents in schools has been widely explored in Western countries, but it is relatively a new research area for Sri Lanka and other South East Asian countries. Researchers' ability to define the extent of the problem is hampered by the lack of a psychometrically sound and culturally validated tool to assess violent behaviors of adolescents in the South East Asian region. Different scales developed in Western settings to measure adolescents' violence are available in the literature. The* Youth Self-Reported Delinquency Scale* and the* Youth Self-Report* have been used to investigate adolescent violence in the Pittsburgh public school system in United States (USA) and these scales assess the antisocial and delinquent behavior [3]. These tools have a limited applicability to measure peer violence in Sri Lanka, as antisocial and criminal behaviors are not commonly identified in the school setting [4, 5]. Violence is a phenomenon with considerable regional and cultural variation and diversity; its conceptualization, definition, and nature may also vary and use of a scale developed in one cultural setting to measure violence may not be directly applicable to another setting [2, 6]. For example, “gun violence” can occur in schools in the United States due to the weapon accessibility [7], but this form of violence may not be common in other settings. Orpinas and Frankowski also developed a self-report tool, the* Aggression Scale*, to measure aggressive behavior among middle school students [8]. It consists of 11 items and focuses mainly on physical aggression, verbal aggression, and emotional arousal that can lead to aggression. The scale measures behaviors that might result in psychological or physical injury to other students. The tool is mainly aimed at assessing aggression. Nonverbal or gestural forms of violence cannot be assessed by using aggression scale. Further it assesses only violence perpetration but not the victimization. Applicability of this tool was restricted since we had reason to believe that nonverbal forms of violence are prevalent in Sri Lankan schools and adolescents are involved in peer violence as perpetrators as well as victims [4, 5]. Another commonly used scale for measuring school violence is Olweus'* Bully/Victim Questionnaire* which targets mainly bullying behavior. The thematic distribution of the questions is mainly focused on measuring verbal bullying and more attention has been paid to identify characteristics of a bully rather than a victim [9]. Thus, its scope was too limited to assess violence in Sri Lankan schools. Gumpel developed a school violence self-report instrument (*School Violence Inventory*) based on Olweus' bully instrument yet focused on the wider construct of school violence and three domains of this phenomenon (direct physical, relational, and sexual) for both perpetrators and their victims [10]. The three types of aggressive behaviors were measured alongside concomitant victimization behavior. This conceptualization allowed examination of both aggressive and victimization typologies simultaneously and enabled the identification of different participant roles: the uninvolved children, pure aggressors, pure victims, and mixed types. Even though the methodology adopted by Gumpel has several strengths, it, too, is not free of limitations to apply in Sri Lankan context. It measures school violence with a selected number of violent acts which have been identified as suitable to assess school violence in schools in Israel and it also included items to assess sexual aggression which is not applicable to Sri Lankan schools. The* Multidimensional School Anger Inventory *(*MSAI*) is another instrument that was designed to assess the affective, cognitive, and behavioral dimension of anger pertinent to the school setting and context [11]. However, when carefully assessing the MSAI scale, it was obvious that the wide spectrum of school violence in Sri Lankan schools cannot be assessed using the MSAI due to its limited scope concentrating mainly on the anger expressions.* Conflict Tactics Scale *(*CTS*) is also a widely used self-report survey in the field of interpersonal violence [12]. The CTS measures physical, verbal, and sexual violence by standard self-report methods and scenario-based self-report methods. This scale is used to measure violence against the partner; thus it has been used by researchers to measure violence in adolescents' dating relationships which is not common in Sri Lankan schools. The* California Healthy Kids Survey *(*CHKS*) is a surveillance instrument administered to students in grades 7, 9, and 11 in California (*N* = 70, 6000) to address multiple victimization experiences of students at school [13]. It can be used to identify subgroups of students who are minimally victimized and chronically victimized. However, CHKS only focuses on victimization of violence, so to also explore perpetration a more comprehensive tool was required. The* Youth Risk Behavior Surveillance System *(*YRBSS*) used by CDC in the United States assesses violence as a part of an overall health behavior assessment of adolescents and measures the prevalence of exposure to a violent incident [14]. This tool was not identified as an appropriate tool to explore wide spectrum of violence among adolescents in Sri Lankan schools as it assessed the behaviors contributing to violence using selected items. Further, YRBSS concentrated on overall youth risk behavior, which contributes to unintentional injuries, violence, suicides, and substance use which did not directly fit with our objective to measure violence among adolescent peers in schools.

Apart from the tools already mentioned, there were others too which were considered for use to assess peer violence among adolescents in Sri Lankan schools but had limited applicability due to different reasons as follows: (i) included only selected violent acts, (ii) did not include verbal or gestural forms of violence, (iii) included items which are culturally irrelevant and inappropriate to measure violence in the local context, (iv) did not distinguish settings for violence exposure, (v) did not identify the perpetrator of violence, (vi) did not identify the entire spectrum of participatory role in violence, and (vii) lacked both empirical validity and test-retest reliability. Existing data suggests that adolescents in the 13–15-year-age group, often referred to as early teenagers, are more prone to involvement in peer violence compared to other students [4, 5]. This project's objective was therefore to develop a valid and a reliable instrument to assess the extent and types of peer violence in schools among 13–15-year-old adolescents in Sri Lanka.

## 2. Methods

**Figure 1:**
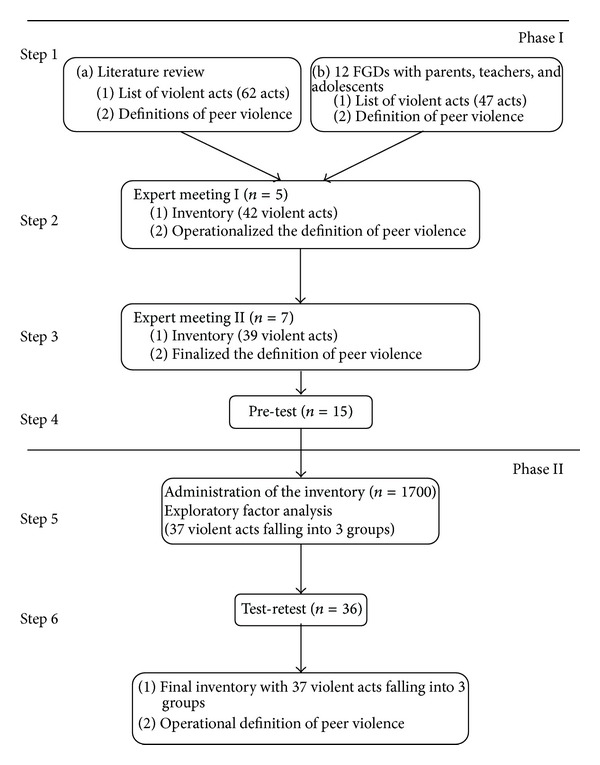
Steps in the development of SLETVI and operationalizing definition of peer violence.

The development of the Sri Lankan Early Teenagers' Violence Inventory (SLETVI) to assess victimization and perpetration of peer violence among 13–15-year-old adolescents was carried out using a combination of qualitative and quantitative methods in a stepwise manner (Figure 1).

*Step  1(a) (literature review).* A literature review focused on identifying operational definitions of peer violence and violent acts that had been used by researchers in other countries and could be adapted to measure peer violence among 13–15-year-old adolescents in Sri Lankan schools was done by searching Medline. Key words included for the literature search were “violence,” “adolescent violence,” “teen violence,” “youth violence,” “school violence,” “peer violence,” “interpersonal violence,” “aggression,” “physical violence,” “verbal violence,” “nonverbal violence,” “relational violence,” “gestural forms of violence,” “electronic aggression,” “bullying,” “cyber-bullying,” “perpetration,” “victimization,” and “scales measuring violence.” Further, unpublished information in the local setting was gathered by referring to the thesis and dissertations available in the libraries and through personal communications. Based on the identified definitions, a draft operational definition to assess peer violence among adolescents in schools was formulated. Based on all the violent acts identified, a list of violent acts which could occur among 13–15-year-old adolescents in schools was prepared.

*Step  1(b) (focus group discussions (FGDs)).* Focus group discussions were then conducted to obtain insight into conceptualizations of peer violence among mid adolescents in the local context, including the identification of different violent acts and terms used to describe them. The FGDs were conducted among 13–15-year-old adolescents in schools, teachers involved in teaching/administration of classes of 13–15-year-olds, and parents of adolescents. The FGDs were conducted in Sinhalese, the local language. Four sessions of FGDs were carried out among 13–15-year-old adolescents, four FGDs among teachers, and another four FGDs among parents in the district of Colombo.

Analysis of FGD results was focused on understanding the conceptualization of 13–15-year-old adolescents' peer violence among each of the different groups included in the FGDs. The important points related to the concept of peer violence were noted to be incorporated into the operational definition. The second objective of the FGDs was to identify the behaviors that the FGD participants viewed as violent acts among 13–15-year-old adolescents. The identified violent behaviors were collated into a list, using local terms.

*Step  2 (operationalizing the definition and developing a tentative inventory of violent acts).* A consultative meeting was arranged with a panel of five experts from community medicine, psychology, school health, primary health care, and education to operationalize the definition of peer violence and to develop a tentative inventory of violent acts, using the information gathered from the literature and the FGDs.

*Step  3 (finalizing the definition and inventory of violent acts).* The tentative operational definition of peer violence developed in first consultative meeting was presented to a panel of seven other experts from community medicine, psychology, school health, primary health care, education, psychiatry, and sociology in a second consultative meeting, and they appraised the validity of the operational definition by judging whether the conceptual definitions had been appropriately translated into operational terms.

The inventory of violent acts formulated was also presented to assess content validity. The panel assessed each item for its appropriateness as a measure of 13–15-year-old adolescents' peer violence, appropriateness of the wording used, and its local relevance. The experts were requested to individually rate each of the violent acts from 1 to 10 for each of the above aspects. The most relevant or appropriate items were rated as “10.” Thereafter, the scores by all experts were added and averaged for each act (Table 1). The decision to retain the violent acts in the inventory was based on whether they achieved a minimum average score of 5.

*Step  4 (pretest)*. The inventory was pretested in a sample of 15 adolescents, five aged 13, five aged 14, and five aged 15 years from three classes in a school in Colombo district to assess how adolescents understood the violent acts listed in the inventory, to check whether the list of violent acts needed revision, and to assess the time required to respond to the inventory. Following the pretest, the students were asked how they understood each listed violent act, to determine whether the wording used was appropriate or whether they would have asked the question in a different way.

*Step  5 (exploratory factor analysis).* This step aimed at assessing the* psychometric properties* of the instrument. The inventory was administered to a total sample of 1700 adolescents (13–15 years old) in 28 randomly chosen public schools in all four educational divisions of Gampaha district. To reduce the number of violent acts and to refine the inventory, exploratory factor analysis using principal component analysis (PCA) was employed on the data obtained by administering the inventory on the study sample. A total of 39 (21 physical and 18 relational) violent acts of victimization and perpetration were included in the inventory. The factor analysis was performed separately for experience of victimization and perpetration. To obtain more interpretable results, factor solution was rotated using Varimax rotation method with Kaiser normalization. Only the violent acts with a loading of greater than 0.4 were accepted as belonging to a particular factor. The criteria used to determine to which factor the violent acts should belong were based on the eigenvalue.

*Step  6 (test-retest)*. Reliability was assessed by readministering the inventory after one week, among 36 randomly selected 13–15-year-old adolescents. These adolescents were also provided with an additional question to indicate whether they had been victims or perpetrators of any violent act during the previous week. Six respondents, who had been involved in violence during previous week, were excluded from the reliability analysis due to concerns that their responses in relation to victimization/perpetration might be distorted. The responses to the violent acts were amalgamated into two categories which indicated whether the adolescent has been involved or not in the violence and reliability was assessed using Cohen's kappa. Thus, reliability of SLETVI was assessed separately to measure victimization and perpetration.

## 3. Results

*Step  1(a) (literature review).* Violence is a concept which is understood differently by different cultures and societies. The occurrence and recognition of violence in a country are dependent on a number of issues at macro- and microlevels. The epidemiological dimensions of violence are not well known or not often discussed openly [15]. Perception of violence would differ from society to society and even from a person to person [6]. The operational definitions related to violence among adolescents in schools in the literature were diverse. The World Health Organization (WHO) has published a working definition for violence which encompasses a broad range of acts [2]. This definition indicates that violence is “intentional use of physical force or power, threatened or actual, against oneself, another person, or a group or community that either results in or has a high likelihood of resulting in injury, death, psychological harm, maldevelopment or deprivation” [2]. A wide spectrum of acts of violence and effects of violence has been captured in this definition. Many recent researches on violence around the world have been based on this definition. The WHO definition on violence highlights the word “intentionality” of the perpetrator irrespective of its outcome. This means even if the desired harm might not happen, if a person performs the act with the intention of harming another person, the action would be identified as violence [2]. Another feature of this definition is the use of the term “power” other than “physical force” to describe the means of violence. This has widened the definition of violence to include violent acts such as threatening, neglecting, and acts of omitting which are acts beyond physical nature. This definition can be considered to cover both overt as well as covert forms of violence. The interpersonal violence among youth has been defined as “the intentional use of physical force or power, threatened or actual, against another person or against a group or community that results in or has a high likelihood of resulting in injury, death, psychological harm, mal development, or deprivation” [16]. Some of the important features of WHO definition, namely, the words power, force, and psychological injuries, are also included in this definition. Olweus defined violence as “an aggressive behavior where the actor or perpetrator uses his or her own body or an object, including a weapon, to inflict injury or discomfort upon another individual” [17]. Thus, violence can take a variety of forms such as physical violence, verbal derogation, or passive obstruction. The effects of violence vary widely and the range can include anything from loss of life or physical injury to emotional harm or wounded pride [18]. The effects even can be a violation of another person's right and freedom of choice, emotional harm, or physical injury. In some communities, emotional harm and violation of rights and freedom of choice may not be taken note of as effects and only physical injuries are used as markers of violence [2].

Violence among adolescents is described in literature under “youth violence” and it refers to harmful behaviors that may commence early and continue into young adulthood [2]. A wide range of violent behaviors such as bullying, slapping, punching, weapon use, rape, and even murder has been shown to occur among youth. Victims have been shown to suffer serious injury, significant social and emotional damage, or even death [2]. Peer violence among adolescents in schools is described in literature under “school violence,” which is considered as a subset of youth violence [2]. School violence is conceptualized as a multifaceted construct that involves any form of violence occurring within schools, among students, among teachers, or between students and teachers and violent acts beyond the physical location and boundaries of the school [19]. According to Hoffman, school violence is “violence involving school children as victims or as a perpetrators [*sic*], in which the setting could be school premises, on the way to school, during school sponsored events, or at group classes of school children held outside the school” [2]. The Centre for the Prevention of School Violence in North Carolina, Department of Juvenile Justice and Delinquency Prevention, defines school violence as “any behavior that violates a school's education mission or climate of respect or jeopardizes the intent of the school to be free of aggression against persons or property, drugs, weapons, disruptions, and disorder” [20]. Hunter and his team defined school violence as “any intentional verbal or physical act producing pain in the recipient of that act while the recipient is under supervision of the school” [21]. This complex nature and variety in the acts of violence have resulted in subcategorization of school violence. Violence by teachers and violence by students are one such classification of school violence.

Gumpel assessed school violence among a sample (*n* = 10,383) of middle and high school students in Israel using School Violence Inventory, which included physical, relational, and sexual aggression [10]. Gumpel identified that verbal and nonverbal or other gestural forms of violence are common among adolescents in schools. The term “relational” was used by Gumpel to collectively describe “verbal and nonverbal” forms of violence. Relational violence is defined as “harming others through purposeful manipulation and damage of their peer relationships” [22]. This form of violence is often observed in bullying behavior. Relational violence can be observed among social groups in which there is purposeful withdrawal of friendship, outright exclusion of others, and spreading of rumors intended to harm another individual within the group [22]. Although there is no consensus within the literature on a universal definition of relational violence, the term is often used interchangeably with social violence, covert violence, indirect violence, and instrumental violence [23].

To summarize, literature reflected that violent acts occurring among adolescent peers can be of diverse nature, that is, physical and relational including sexually related actions. Some studies describe adolescent peer violence in terms of victim versus perpetrator while others described different participatory roles such as pure victim, pure perpetrator, mixed perpetrator-victims, and noninvolved.

The list of violent acts prepared following the literature survey consisted of 56 violent acts (Table 2).

*Step  1(b) (FGDs).* Four FGDs were carried out among 13–15-year-old adolescents: 19 male and 22 females. Four FGDs were carried out including 14 male and 26 female teachers and they were in the 29- to 54-year age group. Their experience in teaching 13–15-year-old adolescents ranged from 4 to 29 years. Four FGDs were carried out among parents: 17 fathers and 21 mothers. Their ages ranged from 35 to 51 years. Four FGDs of each type were enough to reach saturation.

The participants in the FGDs agreed that physical, verbal, and nonverbal or gestural forms of violence were common while sexual violent acts were rare among 13–15-year-old adolescents in Sri Lankan schools. Furthermore, focus group discussions confirmed that all four participatory roles related to adolescent peer violence described in the literature (pure victim, pure perpetrator, aggressive victims, and noninvolved) exist in the local context.

The other focus of the FGD was to get an insight into the behaviors that the FGD participants viewed as violent acts among 13–15-year-old adolescents in Sri Lankan schools, and 47 violent acts were identified (Table 2).

*Step  2 (operationalizing the definition and developing tentative inventory of violent acts).* The first expert panel prepared one tentative inventory of violent acts which included 42 violent acts extracted from the two drafts prepared from the literature review and the FGDs (Table 1). Most of the violent acts mentioned by the FGDs were similar to those mentioned in literature. However, the terms used for some violent acts were found to be different; that is, the term “name calling” used by some authors was renamed as “name calling like* Modaya*,* Bathalee,* and* Gandaya,*” while “spreading rumors using wall posters” was converted to lay terms as “*kalapaththaragaseema*” [5, 24].

Based on the literature review and the FGDs, being a victim to peer violence was defined by the experts as “*being physically or psychologically hurt as a result of a specified violent act committed by a child in his/her school/in another school/in a tuition class.*” Similarly, being a perpetrator of peer violence was defined as “*subjecting a child in his/her school/in another school/in a tuition class to a specified violent act with the intention of hurting him/her physically or psychologically.*”

Furthermore, considering previous research approaches [4, 24] and findings of the FGDs, the five experts advised that it was reasonable to inquire into victimization or perpetration during the period of the previous six months.

*Step  3 (finalizing the definition and inventory of violent acts).* Table 1 shows the assessment by the expert panel of the appropriateness of each violent act in the inventory as a measure of 13–15-year-old adolescents peer violence, appropriateness of the wording used, and relevance in the local context. Most violent acts scored in the range of 8 to 9.7 (out of 10) for all three aspects assessed and 39 violent acts received an average score of 5 or above. Sexual violent acts “trying to kiss you against your will” and “writing sexual graffiti about you” were removed from the final inventory due to low consensual validity.

It was also decided that for each violent act the frequency of victimization and perpetration should be inquired into by specifying four categories: “never,” “once,” “2–5 times,” and “more than 5 times.” This categorization had been used by other researchers, thus making comparisons easy.

*Step  4 (pretest).* A few modifications were made to the tool following the pretest. Wording of two questions was altered to make them more comprehensive. The pretest revealed that it takes 15–20 minutes to respond to the inventory.

*Step  5 (exploratory factor analysis).* One thousand seven hundred early teenagers participated in the validation study. The sample comprised approximately one-third 13-year-olds, one-third 14-year-olds, and one-third 15-year-olds, and 884 (52%) were males and 816 (48%) were females. A large majority were Sinhalese (*n* = 1615, 95.0%) and Buddhists (*n* = 1530, 90%) while 66.9% (*n* = 1138) were from rural settings.

The anti-image correlation matrix revealed that the measure of sampling adequacy for all 39 variables in the inventory was well above the accepted level of 0.5. Bartlett's test of sphericity was significant for both the victimization and perpetration models (*P* < 0.001). The Kaiser-Meyer-Olkin measure was 0.917 for the victimization model and 0.922 for the perpetration model, that is, well above the requirement of 0.6. These confirmed the factorability of data. A factor was considered relevant only if its eigenvalue exceeded 1.0.

The factor loading and grouping of violent acts of perpetration were similar to that of victimization, except for “hitting genitals” which did not successfully load into any domain in the perpetration model. However, it was retained in the final inventory as it had been successfully loaded in the victimization model. There were two violent acts, that is, biting and rumoring using e-mail/internet/Facebook, that were included in the tentative inventory and which were not included in the final inventory as they did not successfully load under any domain. Thus the exploratory factor analysis using PCA reduced the final inventory to 37 violent acts belonging to three dimensions of violence (Table 1). Consistent with previous literature, labels were assigned to each domain. The first domain was named as “*less severe violence*” and it comprised both physical and relational violent acts. Violent acts that had been loaded into second and third domains were identified as “severe” forms. In the second domain only physical violent acts were loaded; thus it was named “*severe physical violence.*” As the third domain was loaded only with relational violent acts, it was named “*severe relational violence.*”

*Step  6 (test-retest)*. The inventory had good test-retest reliability for both victimization and perpetration with a Cohen's kappa coefficient of 0.86 and 0.89, respectively.

## 4. Discussion

SLETVI is designed to be a self-report questionnaire for early teenagers to assess the prevalence of involvement in violence in schools, and we found that it has good psychometric properties. It was developed with a clear conceptualization of the target construct: peer violence. A combination of qualitative methods enabled the generation of items to cover the wide spectrum of violence relevant to the local context. FGD participants and experts agreed that peer violence among 13–15-year-old adolescents in Sri Lankan schools needs to be described in terms of whether an adolescent is a victim or a perpetrator, in terms of the nature of the violent acts (physical or relational), and also in terms of the severity of the violent acts.

The validation study was carried out among a large sample (*n* = 1700) representing all four educational zones in the district of Gampaha. Participants were recruited from 28 schools with varying levels of violence, representing a diverse sample [25]. The factor loading supported the categorization of violent acts according to their severity and other factor analytic studies of violence demonstrate that severe forms of violence load together as do nonsevere violent acts [26]. The reliability assessment used is an accepted method for tools with multi-item scales [27]. The Cohen's kappa thus obtained can be taken as evidence of good reliability.

Compared to the available tools developed in other settings, SLETVI includes culturally acceptable and appropriate violent acts to measure peer violence in Sri Lankan schools. SLETVI assesses the violence with 37 items, which allow describing a wide spectrum of peer violence among 13–15-year-old adolescents in schools. Further, SLETVI is an instrument which could simultaneously assess physical and relational violence among adolescents. Additionally, the list of violent acts included in SLETVI is appropriate to assess both victimization perpetration. Therefore SLETVI can be used to examine the perpetration and victimization of peer violence simultaneously; thus it allows identifying adolescents' participatory role in violence. This would be helpful to tailor intervention programmes targeting each group: “pure victim,” “pure perpetrator,” and “mixed perpetrator victim.” In contrast to other tools, SLETVI specifies the “perpetrator” or “victim” as a “peer adolescent”; thus it specifically measures the violence among adolescent peers in schools. Additionally the setting (schools, tuition classes, school related events, and way to or from school) of the occurrence of violence is clearly indicated in the definition, enabling identifying magnitude of the problem to design prevention programmes specifically based on the setting.

Using a self-report format for gathering information on violence has been shown to be effective and also assures participants the confidentiality of their responses [28]. High rates of literacy as well as acceptable rates of response in previous school based studies using self-administered questionnaires were another reason to select this approach [4, 24]. There is, however, controversy on whether the validity of self-report indices is better or worse than non-self-report indices. Several studies have shown that the construct validity of self-reports is superior to the validity of other measurement approaches [28], and self-reports have the great advantage of easy and inexpensive data collection. Another necessary requirement for accurate self-report is accurate memory. Memory imperfections, such as forward telescoping (i.e., recalling events as having happened more recently than they did) or forgetting, have been recognized as limitations of self-report [29]. According to background information, experts decided that SLETVI should measure violence during the previous six months since it was considered as the optimal recall period which could minimize bias.

*Limitations* of SLETVI should also be noted. Scale development was carried out in Sinhala language as this is the first language among a large majority of adolescents in Gampaha district. Thus, SLETVI should also be validated among adolescents who use Tamil as their first language. Further, SLETVI has been designed to asses peer violence among 13–15-year-old adolescents; using it in other age groups should therefore be done with caution.

*Use of the Inventory.* The systematic use of SLETVI among 13–15-year-old adolescents in schools can be useful for early detection of high risk groups, thereby tailoring school based interventions to prevent violence. Further, in-depth analysis of identified schools with high levels of violence can help to identify priority areas for violence prevention programmes. The inventory can also be used to examine victimization and perpetration of violence simultaneously, and this approach allows identification of different participatory roles in violence with different needs in relation to intervention programs.

## 5. Conclusion

A combination of qualitative and quantitative methods enabled the development of a culturally appropriate operational definition and an inventory, SLETVI, to assess peer violence among 13–15-year-old adolescents in Sri Lankan schools. SLETVI shows good psychometric properties and captures a wide range of violent acts in three categories (less severe violence, severe physical violence, and severe relational violence) among 13–15-year-old adolescents. It is easy to apply and interpret and has good reliability and validity. SLETVI is recommended for measuring peer violence among 13–15-year-old adolescents in schools in Sri Lankan and other South Asian Countries.

## Figures and Tables

**Table 1:** Consensual validity assessed by expert panel for items selected for potential inclusion in the scale and models of PCA developed by exploratory factor analysis carried out on the data obtained by administering inventory among the sample of 1700 adolescents.

Item		Consensual validity assessed by experts			Models of principal component analysis					
		Appropriateness as a measure mean (sd)	Appropriateness of the wording used mean (sd)	Relevance in the local context mean (sd)	Victimization			Perpetration		
					1	2	3	1	2	3
1	Pinching	8.57 (0.53)	9.14 (0.38)	9.29 (0.76)	**0.58**	0.04	0.18	**0.63**	−0.08	0.16
2	Scratching	8.71 (0.95)	9.57 (0.53)	9.71 (0.49)	**0.58**	0.18	0.09	**0.59**	0.14	0.21
3	Pulling hair	8.29 (0.76)	9.29 (0.49)	8.71 (0.76)	**0.54**	0.02	0.15	**0.67**	0.05	0.11
4	Pulling by tie/dress	8.14 (0.69)	9.29 (0.49)	9.29 (0.76)	**0.56**	0.05	0.10	**0.58**	0.041	0.21
5	Pulling ear	8.43 (0.98)	9.14 (0.9)	9.43 (0.53)	**0.57**	0.22	0.14	**0.47**	0.01	0.44
6	Knocking on head	9 00 (0.58)	9.14 (0.38)	9.14 (0.9)	**0.60**	0.08	0.21	**0.59**	0.07	0.35
7	Slapping	8.86 (0.69)	9.14 (0.69)	9.14 (0.38)	**0.67**	0.09	0.15	**0.63**	0.41	0.01
8	Hitting with fist	8.71 (0.76)	8.57 (0.79)	9.29 (0.49)	**0.63**	0.21	0.12	**0.79**	0.17	0.16
9	Slapping ears	8.29 (0.95)	8.86 (0.69)	8.29 (0.95)	**0.57**	0.35	0.09	**0.67**	0.24	0.19
10	Shoving	9.43 (0.53)	9.29 (0.49)	9.57 (0.53)	**0.67**	−0.02	0.18	**0.53**	0.44	−0.04
11	Kicking	8.71 (0.95)	9.29 (0.49)	9.14 (0.69)	**0.61**	0.28	0.10	**0.57**	0.35	0.08
12	Throwing objects	9.57 (0.53)	9.71 (0.49)	9.71 (0.49)	**0.58**	0.06	0.16	**0.73**	0.10	0.14
13	Calling names	9.57 (0.53)	9.29 (0.49)	9.71 (0.49)	**0.45**	−0.18	0.31	**0.50**	−0.01	0.27
14	Excluding from the group	9.29 (0.76)	9.00 (0.00)	9.43 (0.53)	**0.62**	−0.10	0.21	**0.55**	0.14	0.13
15	Teasing or laughing sarcastically	8.43 (0.79)	8.43 (0.53)	8.86 (0.69)	**0.58**	−0.11	0.28	**0.54**	0.22	0.23
16	Not allowing being a member of their group	9.14 (0.69)	9.14 (0.38)	9.43 (0.53)	**0.62**	0.06	0.08	**0.58**	0.36	0.18
17	Not allowing sitting with a friend	8.86 (0.9)	9.14 (0.69)	9.14 (0.9)	**0.59**	0.09	0.11	**0.46**	0.03	0.35
18	Not allowing doing things you like	8.14 (0.69)	8.14 (0.69)	9.00 (0.58)	**0.57**	0.13	0.15	**0.42**	0.15	0.41
19	Not allowing playing with them	9.43 (0.53)	9.43 (0.53)	9.57 (0.53)	**0.56**	0.04	0.26	**0.44**	0.11	0.42
20	Pitting friends against you	9.29 (0.49)	8.86 (0.38)	9.43 (0.53)	**0.64**	0.04	0.19	**0.42**	0.16	**0**.41
21	Tattle-tale teachers to put them against you	8.71 (0.95)	8.71 (0.95)	9.14 (0.69)	**0.59**	0.13	0.22	**0.43**	0.11	0.24
22	Using bad words	9.14 (0.69)	9.00 (0.58)	9.14 (0.69)	**0.55**	0.01	0.43	**0.66**	0.02	0.16
23	Looking down upon	8.57 (0.79)	8.86 (0.9)	9.14 (0.69)	**0.66**	0.14	0.15	**0.41**	0.16	0.02

24	Hitting head against some object	8.14 (0.69)	8.43 (0.53)	9.14 (0.38)	0.40	**0.46**	0.04	0.24	**0.46**	0.21
25	Dragging along the floor	8 00 (0.82)	8.43 (0.53)	8.14 (0.69)	0.16	**0.49**	0.10	0.34	**0.56**	0.06
26	Choking	8.29 (0.49)	8.86 (0.69)	8.71 (0.49)	0.38	**0.44**	0.05	0.33	**0.50**	0.17
27	Burning	8.14 (0.69)	9.14 (0.38)	8.14 (0.90)	0.07	**0.47**	0.09	0.07	**0.58**	−0.02
28	Hitting with a pole	8.86 (0.69)	8.43 (0.53)	9.14 (0.38)	0.24	**0.46**	0.05	0.08	**0.52**	0.37
29	Hitting with sharp weapon	9 00 (0.58)	8.57 (0.53)	8.71 (0.76)	0.12	**0.48**	−0.04	−0.00	**0.70**	0.12
30	Hitting with another weapon	8.86 (0.69)	8.43 (0.53)	9.00 (0.58)	0.11	**0.53**	−0.03	0.13	**0.58**	−0.10
31	Hitting genitals	8.29 (0.76)	8 00 (0.82)	8.29 (0.76)	0.32	**0.41**	0.06	0.29	0.20	0.19

32	Stealing or taking belongings forcefully	8.43 (0.98)	8.71 (0.49)	8.86 (0.69)	0.28	0.14	**0.49**	0.13	0.29	**0.41**
33	Threatening	8.57 (0.79)	8.57 (0.53)	8.57 (0.79)	0.32	0.26	**0.51**	0.30	0.10	**0.67**
34	Telling tales about or spreading rumors using wall posters	8.86 (0.69)	8.43 (0.53)	8.86 (0.69)	0.20	−0.02	**0.57**	0.00	0.19	**0.46**
35	Claiming to involve in an intimate relationship without your interest	8.14 (0.69)	8.14 (0.69)	8.14 (0.38)	0.39	−0.02	**0.41**	0.09	0.17	**0.58**
36	Claiming to continue an intimate relationship without your interest	8.71 (0.95)	8.43 (0.53)	8.43 (0.53)	0.33	−0.08	**0.42**	0.10	0.16	**0.41**
37	Threatening via SMS using mobile phones/phones	8.14 (0.69)	8.43 (0.53)	8.29 (0.76)	0.31	−0.06	**0.43**	0.19	0.10	**0.42**
38	Biting∗	8.29 (0.76)	8.29 (0.49)	8.43 (0.53)	0.22	0.32	0.13	0.34	0.24	−0.03
39	Spreading rumours using e-mail/internet/Facebook∗	8.14 (0.38)	7.71 (0.49)	6.57 (1.13)	0.07	−0.04	0.09	0.13	−0.04	0.17
40	Trying to kiss you against your will∗∗	4.43 (1.72)	5.57 (1.13)	3.43 (0.79)						
41	Threatening to kill∗∗	4.29 (1.11)	4.14 (0.69)	4.00 (0.82)						
42	Writing sexual graffiti about you∗∗	4.71 (1.11)	4.43 (0.79)	3.86 (0.69)						

*Dropped from the final inventory as it did not load in any domain successfully in PCA.

**Dropped from the final inventory due to low consensual validity.

**Items 1–23:**less severe violence. **Items 24**–**31:** severe physical violence. **Items 32**–**37:** severe relational violence.

**Table 2:** List of interpersonal violent activities identified by the literature review* and FGDs^*♣*^.

1	Slapping^∗*♣*^	32	Spreading rumors using wall posters^∗*♣*^
2	Pinching^∗*♣*^	33	Playing mean practical jokes∗
3	Shoving^∗*♣*^	34	Threatening via SMS using mobile phones or phones^∗*♣*^
4	Scratching^∗*♣*^	35	Pulling ear^*♣*^
5	Burning^∗*♣*^	36	Teasing or laughing sarcastically^∗*♣*^
6	Choking^∗*♣*^	37	Threatening to harm^∗*♣*^
7	Biting^∗*♣*^	38	Tattle-tale to teachers to put them against you^∗*♣*^
8	Throwing objects^*♣*^	39	Stealing or taking things forcefully^∗*♣*^
9	Kicking^∗*♣*^	40	Biting^∗*♣*^
10	Knocking on head^∗*♣*^	41	Dragging along the floor^*♣*^
11	Slapping ears^∗*♣*^	42	Not caring^∗*♣*^
12	Hitting with fist^∗*♣*^	43	Not allowing being a member of their group^∗*♣*^
13	Hitting with weapons like a club or pole^∗*♣*^	44	Looking down upon^∗*♣*^
14	Hitting without a weapon^∗*♣*^	45	Not allowing sitting with a friend you like^∗*♣*^
15	Punching^∗*♣*^	46	Not allowing doing things you like^∗*♣*^
16	Calling names^∗*♣*^	47	Not allowing playing with them^∗*♣*^
17	Using bad words^∗*♣*^	48	Pitting friends against you^∗*♣*^
18	Ignoring you^∗*♣*^	49	Cursed you/swore at you^∗*♣*^
19	Forcibly taking belongings^∗*♣*^	50	Spreading rumors using e-mail/Facebook and other social networks^∗*♣*^
20	Pitting friends against you^∗*♣*^	51	Damaged something belongs to you∗
21	Laughing sarcastically/teasing^∗*♣*^	52	Hitting with a sharp weapon^∗*♣*^
22	Pulling by hair^*♣*^	53	Not inviting for gathering^∗*♣*^
23	Pulling by dress, tie^*♣*^	54	Took something belongs to you without the permission
24	Hitting head against some object^∗*♣*^	55	Injured with a gun∗
25	Nonpenetrative or abusive sexual contact∗	56	Drawing graffiti against you∗
26	Hitting genitals^∗*♣*^	57	Threatening to kill∗
27	Intentional touching of the groin∗	58	Try to kiss you against your will∗
28	Not talking^∗*♣*^	59	Forcing sex∗
29	Purposely excluding from the company/deliberately left out from things^∗*♣*^	60	Spreading unpleasant sexual rumors∗
30	Claiming to involve in an intimate relationship without your interest^*♣*^	61	Writing sexual graffiti∗
31	Claiming to continue an intimate relationship without your interest^*♣*^	62	Peeking at someone while he/she was in the toilet or dressing room∗

*Items from literature.

^*♣*^Items from FGDs.
